# Sociodemographic patterns of health insurance coverage in Namibia

**DOI:** 10.1186/s12939-019-0915-4

**Published:** 2019-01-22

**Authors:** Sophie H. Allcock, Elizabeth H. Young, Manjinder S. Sandhu

**Affiliations:** 1 Department of Medicine, University of Cambridge, Cambridge, Cambridgeshire UK; 2 Wellcome Sanger Institute, Wellcome Genome Campus, Hinxton, Cambridgeshire CB10 1SA UK

**Keywords:** Health insurance, Namibia, Education, Women, Wealth

## Abstract

**Introduction:**

Health insurance has been found to increase healthcare utilisation and reduce catastrophic health expenditures in a number of countries; however, coverage is often unequally distributed among populations. The sociodemographic patterns of health insurance in Namibia are not fully understood. We aimed to assess the prevalence of health insurance, the relation between health insurance and health service utilisation and to explore the sociodemographic factors associated with health insurance in Namibia. Such findings may help to inform health policy to improve financial access to healthcare in the country.

**Methods:**

Using data on 14,443 individuals, aged 15 to 64 years, from the 2013 Namibia Demographic and Health Survey, the association between health insurance and health service utilisation was investigated using multivariable mixed effects Poisson regression analyses, adjusted for sociodemographic covariates and regional, enumeration area and household clustering. Multivariable mixed effects Poisson regression analyses were also conducted to explore the association between key sociodemographic factors and health insurance, adjusted for covariates and clustering. Effect modification by sex, education level and wealth quintile was also explored.

**Results:**

Just 17.5% of this population were insured (men: 20.2%; women: 16.2%). In fully-adjusted analyses, education was significantly positively associated with health insurance, independent of other sociodemographic factors (higher education RR: 3.98; 95% CI: 3.11–5.10; *p* < 0.001). Female sex (RR: 0.83; 95% CI: 0.74–0.94; *p* = 0.003) and wealth (highest wealth quintile RR: 13.47; 95% CI: 9.06–20.04; *p* < 0.001) were also independently associated with insurance. There was a complex interaction between sex, education and wealth in the context of health insurance. With increasing education level, women were more likely to be insured (*p* for interaction < 0.001), and education had a greater impact on the likelihood of health insurance in lower wealth quintiles.

**Conclusions:**

In this population, health insurance was associated with health service utilisation but insurance coverage was low, and was independently associated with sex, education and wealth. Education may play a key role in health insurance coverage, especially for women and the less wealthy. These findings may help to inform the targeting of strategies to improve financial protection from healthcare-associated costs in Namibia.

**Electronic supplementary material:**

The online version of this article (10.1186/s12939-019-0915-4) contains supplementary material, which is available to authorized users.

## Introduction

Universal Health Coverage (UHC) is defined by the World Health Organization (WHO) to be where “…all people obtain the health services they need without suffering financial hardship when paying for them” [1]. However, the number of people globally facing catastrophic payments on health is rising [2]. Around 800 million people spend more than 10% of household expenditure on health and around 100 million people are being pushed into extreme poverty every year due to out-of-pocket (OOP) expenditures on health [2].

Namibia is committed to achieving UHC. As an upper-middle income country, with a small population of around 2.5 million people, Namibia’s total health expenditure (THE) as a percentage of gross domestic product (GDP) and per capita health expenditure are comparatively high relative to other sub-Saharan African countries [3–5]. Healthcare in Namibia is funded through Government funding, prepaid private expenditure, OOP expenditure and donor funding [4]. In 2014/15, 64% of THE was provided by the Namibian Government, which equated to around 13% of government expenditure for the fiscal year [4]. Additionally, household expenditures on health fall well below the level indicative of catastrophic health expenditures [6]. However, despite its strong financial position, Namibia may still face challenges to achieving UHC. Namibia experiences substantial wealth inequality across the population [7], which may affect the ability of individuals and households to afford healthcare [8]. Additionally, THE is unevenly distributed, with 36% of THE providing health insurance that covers less than one fifth of the population [4]. Given these inequities in health financing in Namibia, additional financial resources may be needed to realise UHC [4].

Health insurance and other pre-financing mechanisms have been identified as important components of UHC strategies [9–12]. Health insurance has been associated with health-seeking behaviour across sub-Saharan Africa (SSA) and has been found to reduce OOP expenditures, catastrophic spending on health, financial barriers to healthcare, and to protect against poverty in a number of developing countries [13–22]. In Namibia, health insurance has been associated with cancer screening [23–25], timely antenatal care visits and skilled attendance at birth [26, 27], as well as reductions in the economic consequences of HIV-associated health costs [28]. However, the impact of health insurance on health-seeking behaviour more broadly is less well understood in the country. In addition to understanding the coverage of health insurance in a population, it is also important to assess equity in health insurance coverage. Inequalities in Namibia, such as the country’s high income inequality, notable unemployment rate and variable access to and completion of education [7, 29–31], may directly or indirectly impact upon the ability of households to afford healthcare or health insurance. Wealth and education have been widely associated with having insurance in other settings [32–38]; by comparison, the sociodemographic factors associated with health insurance in Namibia have not been well described.

As health insurance is one strategy that could help to achieve UHC, it will be important to assess equity in health insurance coverage across different sociodemographic groups. A better understanding of the sociodemographic factors that are associated with health insurance coverage may help to inform the design and implementation of strategies to improve financial protection from healthcare-associated costs. As such, we first aimed to investigate the relation between health insurance and health service utilisation, and secondly to explore the coverage of health insurance and the demographic factors associated with health insurance in Namibia, using data from the 2013 Namibia Demographic and Health Survey (DHS).

## Methods

### Data sources

To understand the distribution and determinants of health insurance and health service utilisation on a national scale, we used data from the 2013 Namibia DHS. The methods of the 2013 Namibia DHS are described in detail elsewhere [39]. In summary, the DHS included three surveys: the Household Questionnaire, Woman’s Questionnaire and Man’s Questionnaire. Through these surveys, data were collected on 9849 households and 41,646 household members, including in-depth individual data on 10,018 women and 4481 men [39]. The sampling strategy was a two-stage stratified sample design, where stage one involved the selection of enumeration areas (EAs) using stratified proportional size selection [39]. Stage two constituted the random selection of around 20 households within each of the selected EAs [39]. Survey responses were high at over 90% for the Household and Woman’s Questionnaires and 85% for the Man’s Questionnaire [39].

The DHS data are useful for understanding the determinants of health insurance due to the extensive data collected on sociodemographic factors as well as health insurance coverage and health-seeking behaviour (including inpatient and outpatient care). Questions related to health insurance were asked as part of the Woman’s and Man’s Questionnaires. Individuals were asked if they were covered by health insurance and, if so, what type of health insurance they were covered by [39]. Questions related to inpatient and outpatient care seeking were asked to the respondent who answered the Household Questionnaire and included information about the reason for seeking healthcare, the number of visits and the cost of the care.

Education level reflects the highest level of education attended [40], but does not necessarily mean that the level of education was completed. The wealth quintile variable is based on a wealth index factor score, generated using principal components analysis, which was derived from data collected pertaining to household assets, household construction materials and sanitation facilities [40, 41]. Therefore, wealth quintile is a household-level factor, not an individual factor.

### Statistical analyses

All analyses were carried out using Stata 14 software package (StataCorp: College Station, TX, USA). The Household, Woman’s and Man’s datasets were merged and data were cleaned. A subset of 14,443 individuals (9985 women and 4458 men) with information on age, sex, education level, occupation, wealth, residence type, region, marital status and health insurance were included in these analyses. Individuals with occupations classed as “other” were also excluded.

Age was recoded into five-year groups, with those aged 50 to 64 years included in one category. Occupation was recoded into four categories: Professional (including clerical, sales, services), agricultural (including self-employed and employee), manual (including skilled and unskilled) and unemployed. Marital status was recoded to include individuals who were divorced, widowed or no longer living with their partner in the formerly/ever married category. To explore outpatient health seeking behaviour, a variable for whether individuals did or did not seek outpatient care in the four weeks preceding the survey was generated. This was done based on the line number of the individual who sought care. Individuals whose line number matched that of the variable for the line number of the person seeking outpatient care were coded as “1” and those whose line numbers did not match were coded as “0” (not having sought outpatient care). This was repeated for inpatient care. For outpatient care, the variable for the health facility visited was recoded into Government health facilities, private health facilities, other/outreach point /community health worker, pharmacy/shop and traditional healer. For inpatient care the categories were Government health facility, private health facility and “other”.

Categorical data are presented as a frequency and percentage. *P* values were calculated using a chi-squared test for categorical variables. Weighted and unweighted analyses were carried out using DHS sampling weights to assess the representativeness of the results to the whole population. We used the sample weights provided by the Namibia DHS for individual surveys. Unweighted analyses are presented, with weighted results presented in the Additional file 1.

First, the prevalence and distribution of health insurance coverage by sociodemographic characteristics was explored. In supplementary analyses, we investigated health insurance coverage by different insurance types, which included employer-provided, social security, private and “other” insurance, and how this differed by sex.

We then explored health service utilisation as a function of health insurance coverage and other sociodemographic factors. This involved two separate outcomes: whether an individual sought outpatient care in the four weeks preceding the survey; and whether an individual sought inpatient care in the six months preceding the survey. These questions were asked as part of the Household Questionnaire. The household member was identified by a line number; therefore, their health seeking behaviour can be linked to information collected as part of the Woman’s or Man’s Questionnaires. We explored the distribution of individuals who sought inpatient and outpatient care, respectively, by health insurance status and sociodemographic characteristics: health insurance, age, sex, education, wealth, residence type, marital status and occupation. The healthcare provider where care was sought was also explored for both inpatient and outpatient care.

Due to the potential for clustering of exposure and outcome variables at the household, EA and regional level, intraclass correlation coefficients (ICCs) were used to assess clustering of outcome and exposure variables at these levels. ICCs were calculated for each exposure and outcome at the household, EA and regional level and are presented with 95% confidence intervals (95% CIs).

Univariable and multivariable mixed effects Poisson regression analyses were first carried out to explore the association between health insurance and inpatient and outpatient care-seeking, respectively. For both outcomes (sought outpatient care and sought inpatient care), univariable Poisson regression analyses were first carried out (Model 1) to assess the association between the outcomes and health insurance and other potentially confounding sociodemographic factors of interest (age, sex, education, wealth, residence type, marital status and occupation). In Model 2, region, EA and household were included as mixed effects. Finally, in the fully-adjusted multivariable mixed effects model (Model 3), we adjusted for regional, EA and household clustering, and all sociodemographic factors in addition to the primary exposure of interest: health insurance. In mixed effects models, 95% CIs were generated using cluster-robust standard errors.

Multivariable mixed effects Poisson regression analyses were also conducted to explore the sociodemographic factors associated with health insurance. In Model 1 we assessed the univariable association between health insurance and each of the exposures of interest: age, sex, education, wealth, occupation, residence type and marital status. In Model 2, region, EA and household were included as mixed effects. Model 3 was a multivariable mixed effects model which adjusted for all exposures listed above and adjusted for clustering at the regional, EA and household level. In mixed effects models, 95% CIs were generated using cluster-robust standard errors. Effect modification was assessed by stratifying fully-adjusted analyses (Model 3) by sex, education and wealth. We also assessed whether there was statistical evidence of interaction between sex and education, sex and wealth, and education and wealth, in regards to their association with health insurance, using likelihood ratio tests to compare models with and without an interaction term.

## Results

Weighted and unweighted analyses were conducted; here we present unweighted results, with weighted results presented in Additional file 1: Table S1 and Table S2. No material difference was observed between weighted and unweighted results.

### DHS population

Due to survey design, in this subset of 14,443 individuals from the 2013 Namibia DHS, 69.1% were women (Table 1). The population size decreased with increasing age group. The majority of individuals were educated to secondary level (60.2%). The largest proportion of the population was in the fourth wealth quintile (23.4%) and the smallest in the lowest quintile (15.9%). There was an equal distribution by residence type, as to be expected from the study design (urban: 50.9% and rural: 49.1%). This is broadly reflective of Namibia’s population. Most participants were never married (55.0%), with 21.4% currently married and 16.3% living with their partner. Around 50% were unemployed, whilst 35.3% were in professional employment. Similar sociodemographic patterns were observed between men and women (Table 1).

**Table 1:** Distribution of the population by sociodemographic characteristics, stratified by sex

Sociodemographic characteristics	All No. (%)	Men No. (%)	Women No. (%)
Sex			
Men	4458 (30.9)	–	–
Women	9985 (69.1)	–	–
Age group			
15–19	2734 (18.9)	880 (19.7)	1854 (18.6)
20–24	2485 (17.2)	769 (17.3)	1716 (17.2)
25–29	2100 (14.5)	609 (13.7)	1491 (14.9)
30–34	1769 (12.3)	512 (11.5)	1257 (12.6)
35–39	1589 (11.0)	451 (10.1)	1138 (11.4)
40–44	1341 (9.3)	400 (9.0)	941 (9.4)
45–49	1056 (7.3)	308 (6.9)	748 (7.5)
50–64	1369 (9.5)	529 (11.9)	840 (8.4)
Education level			
No education	1213 (8.4)	491 (11.0)	722 (7.2)
Primary	3470 (24.0)	1172 (26.3)	2298 (23.0)
Secondary	8688 (60.2)	2466 (55.3)	6222 (62.3)
Higher	1072 (7.4)	329 (7.4)	743 (7.4)
Wealth quintile			
Lowest	2301 (15.9)	668 (15.0)	1633 (16.4)
Second	2678 (18.5)	861 (19.3)	1817 (18.2)
Middle	3048 (21.1)	1003 (22.5)	2045 (20.5)
Fourth	3381 (23.4)	1036 (23.2)	2345 (23.5)
Highest	3035 (21.0)	890 (20.0)	2145 (21.5)
Residence type			
Urban	7351 (50.9)	2210 (49.6)	5141 (51.5)
Rural	7092 (49.1)	2248 (50.4)	4844 (48.5)
Marital status			
Never married	7947 (55.0)	2628 (59.0)	5319 (53.3)
Currently married	3093 (21.4)	974 (21.9)	2119 (21.2)
Living with partner	2347 (16.3)	678 (15.2)	1669 (16.7)
Formerly/ever married	1056 (7.3)	178 (4.0)	878 (8.8)
Occupation			
Professional	5092 (35.3)	1267 (28.4)	3825 (38.3)
Agricultural	644 (4.5)	442 (9.9)	202 (2.0)
Manual	1435 (9.9)	1063 (23.8)	372 (3.7)
Unemployed	7272 (50.4)	1686 (37.8)	5586 (55.9)
Total	14,443 (100.0)	4458 (100.0)	9985 (100.0)

As expected due to survey design, there was evidence for clustering of health insurance, inpatient care, outpatient care and sociodemographic factors at the household, EA and regional level (Additional file 1: Table S3). Health insurance was clustered at the household and EA level, outpatient and inpatient care were clustered at the household level, education was clustered at the household, and EA level, wealth was clustered at the EA and regional level, residence type was clustered at the regional level and marital status and occupation were clustered at the household level.

### Health insurance coverage

Overall we found that 17.5% of this DHS population had health insurance. A higher proportion of men were insured compared to women (20.2% vs 16.2%) (Table 2). There was a positive relationship between age and health insurance coverage, ranging from 10.0% in those aged 15–19 years to 30.8% in those aged 45–49 years (*p* < 0.001). In these descriptive analyses, the coverage of health insurance increased with levels of education and wealth (*p* < 0.001). We also found that health insurance coverage was notably higher in urban dwellers at 25.7% compared to 8.9% in the rural population (*p* < 0.001). Those who were currently married had the highest coverage of health insurance at 36.8%. As may be expected, health insurance coverage was highest in those in professional employment at 30.8%; however, surprisingly, 7.3% of the unemployed population were insured. The majority of the insured population had employer-provided insurance (54.5%); 29.4% had social security insurance and 21.4% were covered by private insurance (Additional file 1: Figure S1).

**Table 2:** Distribution of health insurance coverage by sociodemographic characteristics (*n* = 14,443)

Sociodemographic characteristics	Health Insurance Coverage No. (%)		
	No	Yes	*p*
Sex			
Men	3556 (79.8)	902 (20.2)	< 0.001
Women	8365 (83.8)	1620 (16.2)	
Age group			
15–19	2462 (90.1)	272 (10.0)	< 0.001
20–24	2220 (89.3)	265 (10.7)	
25–29	1810 (86.2)	290 (13.8)	
30–34	1421 (80.3)	348 (19.7)	
35–39	1254 (78.9)	335 (21.1)	
40–44	988 (73.7)	353 (26.3)	
45–49	731 (69.2)	325 (30.8)	
50–64	1035 (75.6)	334 (24.4)	
Education level			
No education	1165 (96.0)	48 (4.0)	< 0.001
Primary	3257 (93.9)	213 (6.1)	
Secondary	7140 (82.2)	1548 (17.8)	
Higher	359 (33.5)	713 (66.5)	
Wealth quintile			
Lowest	2265 (98.4)	36 (1.6)	< 0.001
Second	2559 (95.6)	119 (4.4)	
Middle	2767 (90.8)	281 (9.2)	
Fourth	2749 (81.3)	632 (18.7)	
Highest	1581 (52.1)	1454 (47.9)	
Residence type			
Urban	5463 (74.3)	1888 (25.7)	< 0.001
Rural	6458 (91.1)	634 (8.9)	
Marital status			
Never married	6988 (87.9)	959 (12.1)	< 0.001
Currently married	1956 (63.2)	1137 (36.8)	
Living with partner	2084 (88.8)	263 (11.2)	
Formerly/ever married	893 (84.6)	163 (15.4)	
Occupation			
Professional	3523 (69.2)	1569 (30.8)	< 0.001
Agricultural	537 (83.4)	107 (16.6)	
Manual	1123 (78.3)	312 (21.7)	
Unemployed	6738 (92.7)	534 (7.3)	
Total	11,921 (82.5)	2522 (17.5)	

*p* value corresponds to a chi-squared test

### Association between health insurance and health service utilisation

To better understand the role of health insurance in health service utilisation, we assessed health insurance as a determinant of utilisation of inpatient (six months prior to the survey) and outpatient care (four weeks prior to the survey). A total of 1355 individuals sought outpatient care in the previous four weeks (9.4%; 7.6% of men and 10.2% of women), whilst 625 individuals sought inpatient care (4.3%; 2.6% of men and 5.1% of women)(Table 3). A higher proportion of those with health insurance sought outpatient and inpatient care compared with the uninsured (*p* < 0.001).

**Table 3:** The distribution of individuals who sought outpatient and inpatient care^a^ by sociodemographic characteristics (*n* = 14,443)

Sociodemographic characteristics	Sought Outpatient care No. (%)			Sought Inpatient care No. (%)		
	No	Yes	*p*	No	Yes	*p*
Health insurance						
No	10,916 (91.6)	1005 (8.4)	< 0.001	11,440 (96.0)	481 (4.0)	< 0.001
Yes	2172 (86.1)	350 (13.9)		2378 (94.3)	144 (5.7)	
Sex						
Men	4119 (92.4)	339 (7.6)	< 0.001	4343 (97.4)	115 (2.6)	< 0.001
Women	8969 (89.8)	1016 (10.2)		9475 (94.9)	510 (5.1)	
Age group						
15–19	2616 (95.7)	118 (4.3)	< 0.001	2670 (97.7)	64 (2.3)	< 0.001
20–24	2338 (94.1)	147 (5.9)		2393 (96.3)	92 (3.7)	
25–29	1913 (91.1)	187 (8.9)		1985 (94.5)	115 (5.5)	
30–34	1579 (89.3)	190 (10.7)		1665 (94.1)	104 (5.9)	
35–39	1424 (89.6)	165 (10.4)		1510 (95.0)	79 (5.0)	
40–44	1178 (87.8)	163 (12.2)		1277 (95.2)	64 (4.8)	
45–49	898 (85.0)	158 (15.0)		1011 (95.7)	45 (4.3)	
50–64	1142 (83.4)	227 (16.6)		1307 (95.5)	62 (4.5)	
Education level						
No education	1111 (91.6)	102 (8.4)	< 0.001	1180 (97.3)	33 (2.7)	0.014
Primary	3133 (90.3)	337 (9.7)		3323 (95.8)	147 (4.2)	
Secondary	7916 (91.1)	772 (8.9)		8300 (95.5)	388 (4.5)	
Higher	928 (86.6)	144 (13.4)		1015 (94.7)	57 (5.3)	
Wealth quintile						
Lowest	2086 (90.7)	215 (9.3)	< 0.001	2207 (95.9)	94 (4.1)	0.878
Second	2446 (91.3)	232 (8.7)		2557 (95.5)	121 (4.5)	
Middle	2795 (91.7)	253 (8.3)		2919 (95.8)	129 (4.2)	
Fourth	3074 (90.9)	307 (9.1)		3227 (95.5)	154 (4.6)	
Highest	2687 (88.5)	348 (11.5)		2908 (95.8)	127 (4.2)	
Residence type						
Urban	6643 (90.4)	708 (9.6)	0.295	7012 (95.4)	339 (4.6)	0.087
Rural	6445 (90.9)	647 (9.1)		6806 (96.0)	286 (4.0)	
Marital status						
Never married	7406 (93.2)	541 (6.8)	< 0.001	7672 (96.5)	275 (3.5)	< 0.001
Currently married	2681 (86.7)	412 (13.3)		2931 (94.8)	162 (5.2)	
Living with partner	2129 (90.7)	218 (9.3)		2211 (94.2)	136 (5.8)	
Formerly/ever married	872 (82.6)	184 (17.4)		1004 (95.1)	52 (4.9)	
Occupation						
Professional	4500 (88.4)	592 (11.6)	< 0.001	4841 (95.1)	251 (4.9)	0.047
Agricultural	580 (90.1)	64 (9.9)		622 (96.6)	22 (3.4)	
Manual	1313 (91.5)	122 (8.5)		1382 (96.3)	53 (3.7)	
Unemployed	6695 (92.1)	577 (7.9)		6973 (95.9)	299 (4.1)	
Total	13,088 (90.6)	1355 (9.4)		13,818 (95.7)	625 (4.3)	

*p* value corresponds to a chi-squared test

^a^Outpatient care sought in four weeks prior to survey and inpatient care sought in six months prior to survey

An equal proportion of insured individuals sought healthcare from private and Government providers for inpatient care (both 49.7%). By contrast, a higher proportion of the uninsured population visited a Government facility for both inpatient and outpatient care, whilst a higher proportion of the insured population visited a private facility for outpatient care (50.0% private; 22.3% Government) (Additional file 1: Figures. S2A and B). We also found that a higher proportion of women sought outpatient care than men (10.2% vs 7.6%, *p* < 0.001) and that the prevalence of seeking outpatient care increased with age (*p <* 0.001), education level (*p* < 0.001) and wealth (*p* < 0.001)(Table 3). A higher proportion of the insured population sought inpatient care compared with the uninsured (5.7% vs 4.0%, *p* < 0.001). The prevalence of inpatient care increased with education level (*p* = 0.014). We did not observe a significant difference in inpatient care by wealth or residence type (*p* > 0.05).

**Fig. 1:**
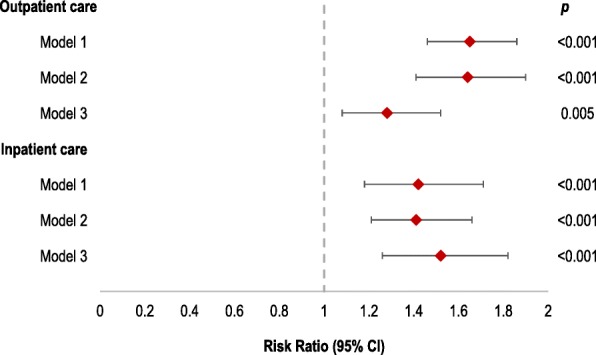
The association between health insurance and inpatient and outpatient care (*n* = 14,443). Model 1: univariable association between health insurance and inpatient and outpatient care, respectively | Model 2: univariable mixed effects model accounting for regional, enumeration area (EA) and household clustering | Model 3: multivariable mixed effects model, accounting for regional, EA and household clustering and adjusting for age, education, wealth, residence type, marital status and occupation | 95% CI: 95% Confidence Interval

To explore the association between health insurance and both outpatient and inpatient care, multivariable mixed effects analyses were conducted to account for clustering and covariates. We found that health insurance was significantly associated with seeking outpatient (Model 3 RR: 1.28; 95% CI: 1.08–1.52; *p* = 0.005) and inpatient care (Model 3 RR: 1.52; 95% CI: 1.26–1.82; *p* < 0.001)(Fig. 1 and Additional file 1: Table S4 and Table S5). This suggests a role for health insurance in health service utilisation. Importantly, women were more likely to seek inpatient and outpatient care, irrespective of insurance status and other sociodemographic factors (Additional file 1: Table S4 and Table S5).

### Sociodemographic determinants of health insurance

As we found an association between health insurance and health service utilisation, we aimed to explore the sociodemographic factors associated with being insured. In multivariable mixed effects Poisson regression analyses (Model 3), women were significantly less likely to be insured than men (RR: 0.83; 95% CI: 0.74–0.94; *p* = 0.003), irrespective of age, education, wealth, residence type, marital status and occupation and clustering (Table 4). Education and wealth were both independently positively associated with health insurance.

**Table 4:** Association between sociodemographic factors and health insurance (*n* = 14,443)

Sociodemographic characteristics	Model 1		Model 2		Model 3	
	RR (95% CI)	*p*	RR (95% CI)	*p*	RR (95% CI)	*p*
Sex						
Men	1.00 (reference)		1.00 (reference)		1.00 (reference)	
Women	0.80 (0.74–0.87)	< 0.001	0.79 (0.71–0.88)	< 0.001	0.83 (0.74–0.94)	0.003
Age group						
15–19	1.00 (reference)		1.00 (reference)		1.00 (reference)	
20–24	1.07 (0.91–1.27)	0.421	0.99 (0.80–1.23)	0.951	0.64 (0.54–0.75)	< 0.001
25–29	1.39 (1.18–1.64)	< 0.001	1.30 (1.00–1.68)	0.049	0.70 (0.60–0.82)	< 0.001
30–34	1.98 (1.69–2.32)	< 0.001	1.80 (1.40–2.32)	< 0.001	0.83 (0.73–0.96)	0.010
35–39	2.12 (1.81–2.49)	< 0.001	1.97 (1.45–2.68)	< 0.001	0.89 (0.75–1.06)	0.200
40–44	2.65 (2.26–3.10)	< 0.001	2.28 (1.67–3.10)	< 0.001	0.98 (0.82–1.16)	0.784
45–49	3.09 (2.63–3.63)	< 0.001	2.66 (1.98–3.57)	< 0.001	1.13 (0.93–1.38)	0.233
50–64	2.45 (2.09–2.88)	< 0.001	2.35 (1.80–3.07)	< 0.001	1.08 (0.87–1.33)	0.503
Education level						
No education	1.00 (reference)		1.00 (reference)		1.00 (reference)	
Primary	1.55 (1.13–2.12)	0.006	1.53 (1.13–2.07)	0.006	1.28 (0.99–1.66)	0.060
Secondary	4.50 (3.38–6.00)	< 0.001	3.44 (2.73–4.34)	< 0.001	2.35 (1.92–2.88)	< 0.001
Higher	16.81 (12.55–22.51)	< 0.001	9.42 (6.14–14.47)	< 0.001	3.98 (3.11–5.10)	< 0.001
Wealth quintile						
Lowest	1.00 (reference)		1.00 (reference)		1.00 (reference)	
Second	2.84 (1.96–4.12)	< 0.001	2.89 (1.76–4.75)	< 0.001	2.52 (1.54–4.13)	< 0.001
Middle	5.89 (4.17–8.34)	< 0.001	6.03 (4.07–8.95)	< 0.001	4.44 (2.90–6.82)	< 0.001
Fourth	11.95 (8.54–16.72)	< 0.001	12.86 (8.97–18.43)	< 0.001	7.58 (5.05–11.39)	< 0.001
Highest	30.62 (22.00–42.62)	< 0.001	30.86 (21.84–43.60)	< 0.001	13.47 (9.06–20.04)	< 0.001
Residence type						
Urban	1.00 (reference)		1.00 (reference)		1.00 (reference)	
Rural	0.35 (0.32–0.38)	< 0.001	0.42 (0.35–0.50)	< 0.001	1.03 (0.90–1.17)	0.676
Marital status						
Never married	1.00 (reference)		1.00 (reference)		1.00 (reference)	
Currently married	3.05 (2.80–3.32)	< 0.001	2.67 (2.20–3.24)	< 0.001	1.68 (1.46–1.93)	< 0.001
Living with partner	0.93 (0.81–1.06)	0.287	1.06 (0.89–1.27)	0.522	1.06 (0.94–1.19)	0.354
Formerly/ever married	1.28 (1.08–1.51)	0.004	1.40 (1.24–1.58)	< 0.001	1.13 (1.04–1.24)	0.005
Occupation						
Professional	1.00 (reference)		1.00 (reference)		1.00 (reference)	
Agricultural	0.54 (0.44–0.66)	< 0.001	0.79 (0.69–0.91)	0.001	0.89 (0.76–1.05)	0.168
Manual	0.71 (0.63–0.80)	< 0.001	0.79 (0.70–0.90)	< 0.001	0.86 (0.78–0.95)	0.003
Unemployed	0.24 (0.22–0.26)	< 0.001	0.32 (0.23–0.44)	< 0.001	0.44 (0.35–0.55)	< 0.001

RR: Risk ratio obtained from Poisson regression analyses | 95% CI: 95% Confidence Intervals

Model 1: univariable association between exposure and having health insurance

Model 2: same as model one with region, enumeration area (EA) and household included as random effects (mixed effects Poisson regression)

Model 3: additionally adjusted for all covariates included in the table (multivariable mixed effects Poisson regression)

**Fig. 2:**
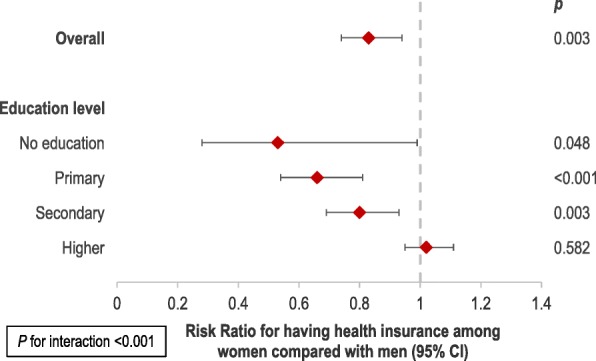
The association between health insurance and sex, stratified by education level. Risk Ratios correspond to the risk of health insurance among women compared with men (reference), overall and stratified by education level | *p* for interaction based on likelihood ratio test comparing models with an without an interaction term | 95% CI: 95% Confidence Interval

**Fig. 3:**
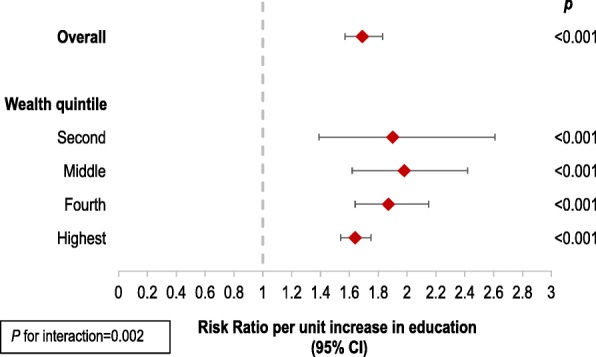
The association between health insurance and education, stratified by wealth quintile. Forest plot showing the greater impact of education on insurance in lower wealth quintiles | Risk ratios correspond to the risk of health insurance per unit increase in education overall and stratified by wealth quintile | *p* for interaction based on likelihood ratio test comparing models with an without an interaction term | 95% CI: 95% Confidence Interval

To further explore the role of sociodemographic factors in health insurance coverage and assess effect modification, we conducted multivariable mixed effects analyses, stratified by sex, education and wealth. When we stratified by sex, we found that education was more strongly associated with health insurance in women than in men (Additional file 1: Table S6). Further, we found that as education level increased, women were more likely to be insured (Fig. 2 and Additional file 1: Table S7). We identified a significant interaction between sex and education (*p* for interaction < 0.001) (Fig. 2 and Additional file 1: Table S7). We also found that wealth modified the association between education and health insurance, with education being more strongly associated with insurance in lower wealth quintiles (*p* for interaction = 0.002)(Fig. 3 and Additional file 1: Table S8). Therefore, education is likely to play a particularly important role in health insurance coverage in less wealthy households. Due to convergence issues, we were unable to stratify by the lowest wealth quintile.

## Discussion

Our findings suggest that health insurance plays a role in healthcare access and health service utilisation in Namibia; however, just 17.5% of this DHS population were insured, leaving a large proportion of the population potentially disadvantaged when accessing healthcare. We found that sex, education and wealth were independently associated with health insurance. Education also modified the association between health insurance and sex and wealth, whereby education was more strongly associated with health insurance in the less wealthy and women. Furthermore, the likelihood of women being insured increased with education level.

There are many factors that may contribute to health insurance coverage; our findings that wealth and education are associated with having health insurance are consistent with those from other settings [32–38]. Wealthier households often have more disposable income to afford insurance. In Namibia, a country with a high income inequality, poorer households can only allocate minor shares of expenditure to healthcare [8]. Furthermore, the structure of many health insurance schemes favours wealthier populations; for example, high annual premiums instead of installment payment options and reimbursement mechanisms, which mean healthcare must first be paid for OOP [42]. The association between sex and health insurance is more complex; by contrast to our findings, studies in Ghana and South Africa identified men to be less likely to have health insurance than women [32, 34]. It has also been suggested that women, as care-givers, are more conscious of the importance of healthcare and insurance and may be more likely to seek healthcare [34, 43]. Similarly, in our analysis, women were more likely to have sought healthcare than men but this health-seeking attitude was not reflected in the patterns of health insurance coverage.

Education, as well as being an independent determinant of health insurance in this Namibian population, also modified sex and wealth disparities in insurance coverage. We found that greater educational attainment increased the likelihood of women being insured. Additionally, when women were educated to higher level there was no difference in insurance compared with men, irrespective of wealth and other sociodemographic factors. This indicates that progression through the education system is especially important for women being insured and is consistent with previous findings that secondary or higher educational attainment is linked to increased coverage of health insurance in other sub-Saharan African populations [35, 36]. We also found that education level was more strongly associated with health insurance in less wealthy populations. Our findings therefore highlight the value and impact of education on health insurance. Education may influence health insurance coverage in a number of ways. Education could improve knowledge and attitudes towards health seeking and the value of health insurance. In Namibia, education has been associated with willingness to join and pay for low-cost health insurance [44] and has also been associated with increased awareness about insurance schemes elsewhere [45, 46]. Therefore, education may empower women and relatively poorer individuals to make choices, including decisions around health [47].

Due to the cross-sectional nature of the data, it was not possible to assess the temporality of the associations observed between sociodemographic factors and health insurance nor between health insurance and health service utilisation. Wealth was also measured at the household level, restricting our understanding of the effects of individual wealth on health insurance. Other factors beyond the scope of this analysis may also influence health service utilisation or health insurance coverage, such as the likelihood that the consumer will become ill, preexisting medical conditions, or individual knowledge, attitudes and practice towards health and insurance [42, 48–50]. It was also not possible to explore the willingness to pay for health insurance. A further limitation is that the data used for these analyses were collected in 2013, and thus may not fully reflect the situation in Namibia at present.

Education and public engagement have been identified as key strategies for the uptake and acceptability of health insurance in other settings [49, 51]. Improving access to, and the quality of, education is an important component of multiple government strategies in Namibia [29, 52, 53] and our findings further highlight the importance of the country’s commitments to improving education. Although access to education in Namibia is high overall, including for women, attendance and the quality of education is variable and often inadequate in lower-income communities, marginalised populations and in remote or rural areas [29]. Whilst a high percentage of the Namibian population complete primary education, transition to and completion of secondary and higher education could be improved [29]. Our findings suggest that improvements in access to education may help individuals to better manage their health but further research is needed to better understand this relationship. These findings have implications for the design and implementation of strategies to scale-up health insurance coverage or improve financial protection for more vulnerable populations. Health insurance could be scaled-up through community engagement strategies that utilise the media and other advocacy tools [54, 55]. Furthermore, mechanisms to make health insurance more affordable through subsidisation, for example, may help to increase uptake [12, 44]. Employer-provided schemes, which accounted for more than half of insurance in this population, could be expanded to the informal sector. For example, one study in Namibia found that employers on commercial farms were receptive to providing a co-pay insurance plan for their employees [56]. An alternative solution in countries like Namibia, where around a fifth of healthcare is financed via private health insurance, is that Governments could target public financing to populations less able or likely to participate in voluntary insurance schemes [57].

## Conclusions

In conclusion, health insurance is an important component of health service utilisation in Namibia, but inequities in the coverage of these insurance schemes means that many individuals could be at a disadvantage when accessing healthcare. Specifically, women and those with lower levels of education and wealth were less likely to be covered by health insurance. Our findings suggest that, in Namibia, education may be important for bridging gaps in health insurance coverage for women and the less wealthy, but further research is needed to fully understand this relationship. These findings could inform the design and implementation of interventions to scale-up health insurance or provide greater financial protection from healthcare-associated costs for uninsured populations. Additional research is also needed to evaluate the effectiveness of insurance schemes and the quality of care received as a result of being insured in Namibia and elsewhere if UHC is to be realised.

## Additional file

Additional file 1: **Figure S1.** Proportion of insured individuals with each type of health insurance, stratified by sex. Number labels correspond to the number of individuals. **Figure S2.** Type of healthcare provider where inpatient and outpatient care was sought by health insurance coverage | A insured *n* = 350 uninsured *n* = 1005; B insured *n* = 143 uninsured *n* = 479 | HF: health facility | OP: outreach point | CHW: community health worker. **Table S1.** Weighted prevalence of health insurance by sociodemographic factors. **Table S2.** Weighted prevalence of seeking outpatient and inpatient care* by sociodemographic characteristics. **Table S3.** Clustering of sociodemographic factors within households, EAs and regions (*n* = 14,443). **Table S4.** Association between exposures of interest and seeking outpatient care in the four weeks prior to the survey (*n* = 14,443). **Table S5.** Association between exposures of interest and inpatient care (*n* = 14,443). **Table S6.** Association between sociodemographic factors and health insurance, stratified by sex. **Table S7.** Association between sociodemographic factors and health insurance, stratified by education level. **Table S8.** Association between sociodemographic factors and health insurance, stratified by wealth quintile. (DOCX 178 kb)

## Abbreviations

DHS: Demographic and Health Survey
EA: enumeration area
GDP: Gross Domestic Product
OOP: out-of-pocket
SD: standard deviation
SSA: sub-Saharan Africa
THE: Total Health Expenditure
UHC: Universal Health Coverage
WHO: World Health Organization
