# Supplementary material for: Sociodemographic patterns of health insurance coverage in Namibia
Source: Int J Equity Health. 2019 Jan 22;18:16. doi: 10.1186/s12939-019-0915-4 (PMC6341740; doi:10.1186/s12939-019-0915-4)
Supplement: Supplementary file 1 — Figure S1. Proportion of insured individuals with each type of health insurance, stratified by sex. Number labels correspond to the number of individuals. Figure S2. Type of healthcare provider where inpatient and outpatient care was sought by health insurance coverage | A insured n = 350 uninsured n = 1005; B insured n = 143 uninsured n = 479 | HF: health facility | OP: outreach point | CHW: community health worker. Table S1. Weighted prevalence of health insurance by sociodemographic factors. Table S2. Weighted prevalence of seeking outpatient and inpatient care* by sociodemographic characteristics. Table S3. Clustering of sociodemographic factors within households, EAs and regions (n = 14,443). Table S4. Association between exposures of interest and seeking outpatient care in the four weeks prior to the survey (n = 14,443). Table S5. Association between exposures of interest and inpatient care (n = 14,443). Table S6. Association between sociodemographic factors and health insurance, stratified by sex. Table S7. Association between sociodemographic factors and health insurance, stratified by education level. Table S8. Association between sociodemographic factors and health insurance, stratified by wealth quintile. (DOCX 178 kb) [file 12939_2019_915_MOESM1_ESM.docx]

**Additional file 1**

**Figure S1** Proportion of insured individuals with each type of health insurance, stratified by sex. Number labels correspond to the number of individuals.


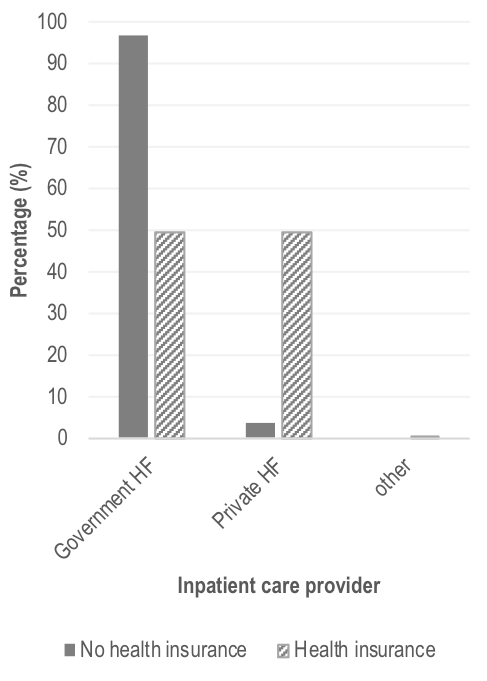

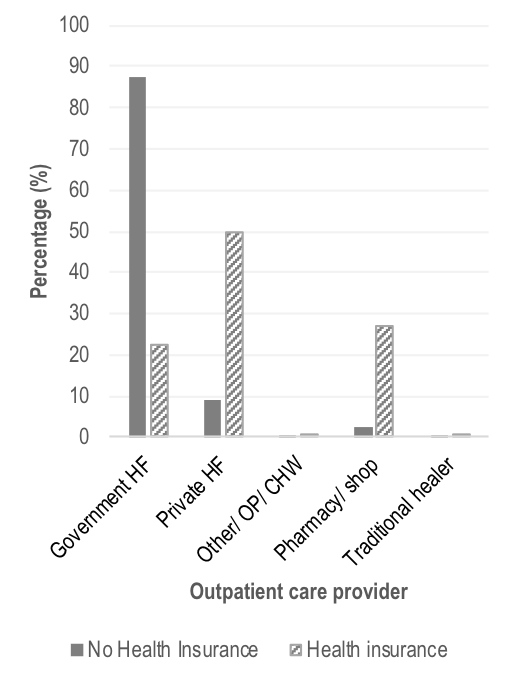


**A**

**B**

**Figure S2** Type of healthcare provider where inpatient and outpatient care was sought by health insurance coverage | **A** insured n=350 uninsured n=1,005; **B** insured n=143 uninsured n=479 | HF: health facility | OP: outreach point | CHW: community health worker.

**Table S1** Weighted prevalence of health insurance by sociodemographic factors

| **Sociodemographic characteristics** | **Health Insurance Coverage**  No. (%) | |
| --- | --- | --- |
|  | **No** | **Yes** |
| **Sex** |  |  |
| Men | 3,510 (78.7) | 952 (21.3) |
| Women | 7,529 (82.4) | 1,610 (17.6) |
| **Age group** |  |  |
| 15-19 | 2,484 (88.1) | 337 (11.9) |
| 20-24 | 2,256 (87.2) | 331 (12.8) |
| 25-29 | 1,811 (84.6) | 329 (15.4) |
| 30-34 | 1,397 (78.9) | 374 (21.1) |
| 35-39 | 1,207 (78.9) | 339 (21.9) |
| 40-44 | 926 (71.8) | 364 (28.2) |
| 45-49 | 662 (66.8) | 329 (33.2) |
| 50-64 | 297 (65.0) | 160 (35.0) |
| **Education level** |  |  |
| No education | 774 (95.1) | 40 (4.9) |
| Primary | 2,761 (95.0) | 147 (5.1) |
| Secondary | 6,998 (81.9) | 1,551 (18.1) |
| Higher | 507 (38.1) | 825 (61.9) |
| **Wealth quintile** |  |  |
| Lowest | 2,044 (98.4) | 33 (1.6) |
| Second | 2355 (95.8) | 103 (4.2) |
| Middle | 2,482 (89.6) | 288 (10.4) |
| Fourth | 2,507 (80.2) | 620 (19.8) |
| Highest | 1,651 (52.1) | 1,518 (47.9) |
| **Residence type** |  |  |
| Urban | 5,584 (73.0) | 2,062 (27.0) |
| Rural | 5,455 (91.6) | 500 (8.4) |
| **Marital status** |  |  |
| Never married | 7,105 (86.4) | 1,118 (13.6) |
| Currently married | 1,484 (58.6) | 1,048 (41.4) |
| Living with partner | 1,834 (87.6) | 259 (12.4) |
| Formerly/ever married | 616 (81.9) | 137 (18.1) |
| **Occupation** |  |  |
| Professional | 3,477 (68.5) | 1,603 (31.8) |
| Agricultural | 338 (84.1) | 64 (15.9) |
| Manual | 1,115 (78.4) | 308 (21.7) |
| Unemployed | 6,108 (91.2) | 587 (8.8) |
|  |  |  |
| **Total** | 11,039 (81.2) | 2,561 (18.8) |

Number and percentage estimates generated using DHS survey weight |

Due to rounding, some totals may not correspond with the sum of the separate figures in the table

**Table S2** Weighted prevalence of seeking outpatient and inpatient care* by sociodemographic characteristics

| **Sociodemographic characteristics** | **Sought Outpatient care**  No. (%) | | **Sought Inpatient care**  No. (%) | |
| --- | --- | --- | --- | --- |
|  | **No** | **Yes** | **No** | **Yes** |
| **Health insurance** |  |  |  |  |
| No | 10,173 (92.2) | 866 (7.9) | 10,619 (96.2) | 419 (3.8) |
| Yes | 2,244 (87.6) | 317 (12.4) | 2,418 (94.4) | 144 (5.6) |
| **Sex** |  |  |  |  |
| Men | 4,130 (92.6) | 331 (7.4) | 4,348 (97.5) | 113 (2.5) |
| Women | 8,287 (90.7) | 853 (9.3) | 8,689 (95.1) | 450 (4.9) |
| **Age group** |  |  |  |  |
| 15-19 | 2,693 (95.5) | 128 (4.5) | 2,766 (98.1) | 54 (1.9) |
| 20-24 | 2,433 (94.0) | 155 (6.0) | 2,500 (96.6) | 87 (3.4) |
| 25-29 | 1,967 (91.9) | 173 (8.1) | 2,030 (94.9) | 110 (5.1) |
| 30-34 | 1,576 (89.0) | 195 (11.0) | 1,661 (93.8) | 110 (6.2) |
| 35-39 | 1,385 (89.6) | 161 (10.4) | 1,462 (94.6) | 84 (5.5) |
| 40-44 | 1,127 (87.4) | 162 (14.3) | 1,232 (95.5) | 58 (4.5) |
| 45-49 | 848 (85.7) | 142 (14.3) | 949 (95.8) | 41 (4.2) |
| 50-64 | 389 (85.0) | 68 (15.0) | 437 (95.7) | 20 (4.3) |
| **Education level** |  |  |  |  |
| No education | 754 (92.8) | 59 (7.2) | 792 (97.4) | 21 (2.6) |
| Primary | 2,653 (91.2) | 255 (8.8) | 2,790 (96.0) | 117 (4.0) |
| Secondary | 7,826 (91.5) | 723 (8.5) | 8,188 (95.8) | 360 (4.2) |
| Higher | 1,184 (88.9) | 147 (11.1) | 1,267 (95.2) | 64 (4.8) |
| **Wealth quintile** |  |  |  |  |
| Lowest | 1,905 (91.8) | 171 (8.3) | 1,996 (96.1) | 81 (3.9) |
| Second | 2,257 (91.8) | 201 (8.2) | 2,352 (95.7) | 106 (4.3) |
| Middle | 2,553 (92.1) | 218 (7.9) | 2,662 (96.1) | 109 (3.9) |
| Fourth | 2,863 (91.6) | 264 (8.4) | 2,973 (95.1) | 153 (4.9) |
| Highest | 2,839 (89.6) | 330 (10.4) | 3,054 (96.4) | 115 (3.6) |
| **Residence type** |  |  |  |  |
| Urban | 6,958 (91.0) | 688 (9.0) | 7,318 (95.7) | 328 (4.3) |
| Rural | 5,459 (91.7) | 495 (8.3) | 5,719 (96.1) | 235 (4.0) |
| **Marital status** |  |  |  |  |
| Never married | 7,659 (93.1) | 564 (6.9) | 7,949 (96.7) | 274 (3.3) |
| Currently married | 2,218 (87.6) | 313 (12.4) | 2,388 (94.3) | 143 (5.7) |
| Living with partner | 1,907 (91.1) | 187 (8.9) | 1,979 (94.5) | 114 (5.5) |
| Formerly/ ever married | 633 (84.1) | 120 (15.9) | 722 (95.9) | 31 (4.1) |
| **Occupation** |  |  |  |  |
| Professional | 4,519 (89.0) | 561 (11.0) | 4,822 (94.9) | 258 (5.1) |
| Agricultural | 364 (90.5) | 38 (9.5) | 390 (97.1) | 12 (2.9) |
| Manual | 1,310 (92.1) | 113 (8.0) | 1,379 (96.9) | 44 (3.1) |
| Unemployed | 6,223 (93.0) | 471 (7.0) | 6,446 (96.3) | 249 (3.7) |
| **Total** | **12,417 (91.3)** | **1,183 (8.7)** | **13,038 (95.9)** | **563 (4.1)** |

*Outpatient care sought in four weeks prior to survey and inpatient care sought in six months prior to survey | number and percentage estimates generated using DHS survey weight | Due to rounding, some totals may not correspond with the sum of the separate figures in the table

**Table S3** Clustering of sociodemographic factors within households, EAs and regions (n=14,443)

|  | **Household** | **EA** | **Region** |
| --- | --- | --- | --- |
|  | **ICC (95% CI)** | **ICC (95% CI)** | **ICC (95% CI)** |
|  |  |  |  |
| **Health insurance** | 0.35 (0.33 - 0.37) | 0.21 (0.18 - 0.23) | 0.05 (0.01 - 0.09) |
|  |  |  |  |
| **Outpatient care** | 0.13 (0.10 - 0.15) | 0.02 (0.01 - 0.02) | 0.00 (0.00 - 0.00) |
|  |  |  |  |
| **Inpatient care** | 0.15 (0.12 - 0.17) | 0.00 (0.00 - 0.01) | 0.00 (0.00 - 0.00) |
|  |  |  |  |
| **Sex** | 0.00 (0.00 - 0.03) | 0.02 (0.01 - 0.02) | 0.00 (0.00 - 0.00) |
|  |  |  |  |
| **Age** | 0.00 (0.00 - 0.03) | 0.03 (0.02 - 0.04) | 0.01 (0.00 - 0.02) |
|  |  |  |  |
| **Education** | 0.38 (0.36 - 0.40) | 0.23 (0.21 - 0.26) | 0.10 (0.03 - 0.18) |
|  |  |  |  |
| **Wealth quintile** | N/A | 0.69 (0.67 - 0.72) | 0.31 (0.14 - 0.48) |
|  |  |  |  |
| **Residence type** | N/A | N/A | 0.35 (0.17 - 0.53) |
|  |  |  |  |
| **Marital status** | 0.19 (0.17 - 0.22) | 0.07 (0.06 - 0.09) | 0.05 (0.01 - 0.08) |
|  |  |  |  |
| **Occupation** | 0.17 (0.14 - 0.19) | 0.11 (0.09 - 0.12) | 0.05 (0.01 - 0.09) |
|  |  |  |  |

N/A where wealth and residence type are household or EA level factors | EA: enumeration area | ICC: intraclass correlation coefficient | 95% CI: 95% confidence interval

**Table S4** Association between exposures of interest and seeking outpatient care in the four weeks prior to the survey (n=14,443)

| **Exposures of interest** | **Model 1** | | **Model 3** | |
| --- | --- | --- | --- | --- |
|  | **RR (95% CI)** | ***p*** | **RR (95% CI)** | ***p*** |
| **Health Insurance** |  |  |  |  |
| No | 1.00 (reference) |  | 1.00 (reference) |  |
| Yes | 1.65 (1.46 – 1.86) | <0.001 | 1.28 (1.08 – 1.52) | 0.005 |
| **Sex** |  |  |  |  |
| Men | 1.00 (reference) |  | 1.00 (reference) |  |
| Women | 1.34 (1.18 – 1.51) | <0.001 | 1.31 (1.14 – 1.50) | <0.001 |
| **Age group** |  |  |  |  |
| 15-19 | 1.00 (reference) |  | 1.00 (reference) |  |
| 20-24 | 1.37 (1.08 – 1.75) | 0.011 | 1.30 (1.01 – 1.69) | 0.046 |
| 25-29 | 2.06 (1.64 – 2.60) | <0.001 | 1.87 (1.38 – 2.54) | <0.001 |
| 30-34 | 2.49 (1.98 – 3.13) | <0.001 | 2.14 (1.77 – 2.60) | <0.001 |
| 35-39 | 2.41 (1.90 – 3.05) | <0.001 | 2.02 (1.57 – 2.59) | <0.001 |
| 40-44 | 2.82 (2.22 – 3.57) | <0.001 | 2.25 (1.69 – 3.01) | <0.001 |
| 45-49 | 3.47 (2.73 – 4.40) | <0.001 | 2.69 (2.10 – 3.45) | <0.001 |
| 50-64 | 3.84 (3.08 – 4.80) | <0.001 | 3.15 (2.37 – 4.19) | <0.001 |
| **Education level** |  |  |  |  |
| No education | 1.00 (reference) |  | 1.00 (reference) |  |
| Primary | 1.16 (0.93 – 1.44) | 0.202 | 1.28 (1.10 – 1.48) | 0.002 |
| Secondary | 1.06 (0.86 – 1.30) | 0.601 | 1.32 (1.12 – 1.54) | 0.001 |
| Higher | 1.60 (1.24 – 2.06) | <0.001 | 1.46 (1.11 – 1.94) | 0.008 |
| **Wealth quintile** |  |  |  |  |
| Lowest | 1.00 (reference) |  | 1.00 (reference) |  |
| Second | 0.93 (0.77 – 1.12) | 0.424 | 0.94 (0.75 – 1.17) | 0.558 |
| Middle | 0.89 (0.74 – 1.07) | 0.202 | 0.87 (0.72 – 1.05) | 0.146 |
| Fourth | 0.97 (0.82 – 1.16) | 0.748 | 0.90 (0.70 – 1.15) | 0.393 |
| Highest | 1.23 (1.04 – 1.45) | 0.018 | 0.97 (0.74 – 1.28) | 0.836 |
| **Residence type** |  |  |  |  |
| Urban | 1.00 (reference) |  | 1.00 (reference) |  |
| Rural | 0.95 (0.85 – 1.05) | 0.319 | 0.99 (0.80 – 1.23) | 0.952 |
| **Marital status** |  |  |  |  |
| Never married | 1.00 (reference) |  | 1.00 (reference) |  |
| Currently married | 1.96 (1.72 – 2.22) | <0.001 | 1.17 (0.98 – 1.40) | 0.081 |
| Living with partner | 1.36 (1.17 – 1.60) | <0.001 | 1.12 (0.95 – 1.34) | 0.188 |
| Formerly/ ever married | 2.56 (2.17 – 3.03) | <0.001 | 1.59 (1.28 – 1.98) | <0.001 |
| **Occupation** |  |  |  |  |
| Professional | 1.00 (reference) |  | 1.00 (reference) |  |
| Agricultural | 0.86 (0.66 – 1.11) | 0.233 | 1.00 (0.84 – 1.18) | 0.949 |
| Manual | 0.73 (0.60 – 0.89) | 0.002 | 0.89 (0.77 – 1.02) | 0.089 |
| Unemployed | 0.68 (0.61 – 0.77) | <0.001 | 0.93 (0.85 – 1.02) | 0.099 |

RR: Risk Ratio | 95% CI: 95% Confidence Interval | Model 1: Univariable model | Model 3: Adjusted for regional, enumeration area and household clustering and all other covariates in the table | Estimates for Model 2, as described in methods, not shown

**Table S5** Association between exposures of interest and inpatient care (n=14,443)

| **Exposures of interest** | **Model 1** | | **Model 3** | |
| --- | --- | --- | --- | --- |
|  | **RR (95% CI)** | ***p*** | **RR (95% CI)** | ***p*** |
| **Health Insurance** |  |  |  |  |
| No | 1.00 (reference) |  | 1.00 (reference) |  |
| Yes | 1.42 (1.18 – 1.71) | <0.001 | 1.52 (1.26 – 1.82) | <0.001 |
| **Sex** |  |  |  |  |
| Men | 1.00 (reference) |  | 1.00 (reference) |  |
| Women | 1.98 (1.62 – 2.42) | <0.001 | 1.95 (1.55 – 2.45) | <0.001 |
| **Age group** |  |  |  |  |
| 15-19 | 1.00 (reference) |  | 1.00 (reference) |  |
| 20-24 | 1.58 (1.15 – 2.18) | 0.005 | 1.50 (1.07 – 2.10) | 0.018 |
| 25-29 | 2.34 (1.72 – 3.18) | <0.001 | 2.09 (1.54 – 2.83) | <0.001 |
| 30-34 | 2.51 (1.84 – 3.43) | <0.001 | 2.19 (1.40 – 3.43) | 0.001 |
| 35-39 | 2.12 (1.53 – 2.95) | <0.001 | 1.82 (1.13 – 2.94) | 0.014 |
| 40-44 | 2.04 (1.44 – 2.88) | <0.001 | 1.77 (1.29 – 2.44) | <0.001 |
| 45-49 | 1.82 (1.24 – 2.67) | 0.002 | 1.58 (1.11 – 2.25) | 0.011 |
| 50-64 | 1.94 (1.36 – 2.74) | <0.001 | 1.88 (1.24 – 2.86) | 0.003 |
| **Education level** |  |  |  |  |
| No education | 1.00 (reference) |  | 1.00 (reference) |  |
| Primary | 1.56 (1.07 – 2.27) | 0.022 | 1.70 (1.05 – 2.75) | 0.031 |
| Secondary | 1.64 (1.15 – 2.34) | 0.006 | 1.82 (1.11 – 2.97) | 0.017 |
| Higher | 1.95 (1.27 – 3.00) | 0.002 | 1.96 (1.13 – 3.40) | 0.017 |
| **Wealth quintile** |  |  |  |  |
| Lowest | 1.00 (reference) |  | 1.00 (reference) |  |
| Second | 1.11 (0.85 – 1.45) | 0.464 | 1.06 (0.86 – 1.31) | 0.590 |
| Middle | 1.04 (0.79 – 1.35) | 0.794 | 0.92 (0.70 – 1.20) | 0.538 |
| Fourth | 1.12 (0.86 – 1.44) | 0.406 | 0.88 (0.69 – 1.13) | 0.321 |
| Highest | 1.02 (0.79 – 1.34) | 0.860 | 0.67 (0.51 – 0.88) | 0.004 |
| **Residence type** |  |  |  |  |
| Urban | 1.00 (reference) |  | 1.00 (reference) |  |
| Rural | 0.87 (0.75 – 1.02) | 0.095 | 0.87 (0.70 – 1.09) | 0.236 |
| **Marital status** |  |  |  |  |
| Never married | 1.00 (reference) |  | 1.00 (reference) |  |
| Currently married | 1.51 (1.25 – 1.84) | <0.001 | 1.21 (1.00 – 1.47) | 0.048 |
| Living with partner | 1.68 (1.36 – 2.06) | <0.001 | 1.41 (1.10 – 1.81) | 0.006 |
| Formerly/ ever married | 1.42 (1.06 – 1.91) | 0.020 | 1.15 (0.87 – 1.52) | 0.339 |
| **Occupation** |  |  |  |  |
| Professional | 1.00 (reference) |  | 1.00 (reference) |  |
| Agricultural | 0.69 (0.45 – 1.07) | 0.099 | 1.01 (0.60 – 1.69) | 0.975 |
| Manual | 0.75 (0.56 – 1.01) | 0.056 | 1.06 (0.79 – 1.41) | 0.715 |
| Unemployed | 0.83 (0.71 – 0.99) | 0.034 | 1.11 (0.97 – 1.28) | 0.141 |

RR: Risk Ratio | 95% CI: 95% Confidence Interval | Model 1: Univariable model | Model 3: Adjusted for regional, enumeration area and household clustering and all other covariates in the table | Estimates for Model 2, as described in methods, not shown

**Table S6** Association between sociodemographic factors and health insurance, stratified by sex

| **Sociodemographic characteristics** | **Men** | | **Women** | | ***p* for interaction** |
| --- | --- | --- | --- | --- | --- |
|  | **RR (95% CI)** | ***p*** | **RR (95% CI)** | ***p*** |  |
| **Age group** |  |  |  |  |  |
| 15-19 | 1.00 (reference) |  | 1.00 (reference) |  |  |
| 20-24 | 0.63 (0.49 – 0.81) | 0.001 | 0.63 (0.51 – 0.79) | <0.001 |  |
| 25-29 | 0.80 (0.60 – 1.06) | 0.118 | 0.65 (0.54 – 0.79) | <0.001 |  |
| 30-34 | 0.88 (0.70 – 1.10) | 0.252 | 0.80 (0.65 – 0.98) | 0.033 |  |
| 35-39 | 0.92 (0.70 – 1.22) | 0.577 | 0.88 (0.72 – 1.07) | 0.190 |  |
| 40-44 | 1.04 (0.76 – 1.43) | 0.809 | 0.94 (0.78 – 1.13) | 0.506 |  |
| 45-49 | 1.17 (0.89 – 1.54) | 0.269 | 1.09 (0.87 – 1.37) | 0.457 |  |
| 50-64 | 1.10 (0.82 – 1.49) | 0.509 | 1.08 (0.87 – 1.35) | 0.481 |  |
| **Education level** |  |  |  |  |  |
| No education | 1.00 (reference) |  | 1.00 (reference) |  | <0.001 |
| Primary | 1.25 (0.90 – 1.75) | 0.184 | 1.48 (0.91 – 2.41) | 0.118 |  |
| Secondary | 1.93 (1.38 – 2.71) | <0.001 | 3.19 (2.29 – 4.44) | <0.001 |  |
| Higher | 2.77 (1.97 – 3.91) | <0.001 | 5.83 (3.98 – 8.55) | <0.001 |  |
| **Wealth quintile** |  |  |  |  |  |
| Lowest | 1.00 (reference) |  | 1.00 (reference) |  | 0.012 |
| Second | 4.17 (2.08 – 8.38) | <0.001 | 1.89 (1.16 – 3.10) | 0.011 |  |
| Middle | 6.84 (3.70 – 12.62) | <0.001 | 3.59 (2.15 – 5.99) | <0.001 |  |
| Fourth | 11.09 (5.86 – 21.01) | <0.001 | 6.33 (3.79 – 10.57) | <0.001 |  |
| Highest | 18.32 (9.35 – 35.91) | <0.001 | 11.75 (7.38 – 18.72) | <0.001 |  |
| **Residence type** |  |  |  |  |  |
| Urban | 1.00 (reference) |  | 1.00 (reference) |  |  |
| Rural | 0.93 (0.77 – 1.12) | 0.462 | 1.11 (0.96 – 1.28) | 0.178 |  |
| **Marital status** |  |  |  |  |  |
| Never married | 1.00 (reference) |  | 1.00 (reference) |  |  |
| Currently married | 1.55 (1.35 – 1.79) | <0.001 | 1.74 (1.50 – 2.02) | <0.001 |  |
| Living with partner | 1.40 (1.17 – 1.68) | <0.001 | 0.82 (0.72 – 0.94) | 0.005 |  |
| Formerly/ ever married | 1.14 (0.86 – 1.51) | 0.377 | 1.14 (1.02 – 1.27) | 0.026 |  |
| **Occupation** |  |  |  |  |  |
| Professional | 1.00 (reference) |  | 1.00 (reference) |  |  |
| Agricultural | 0.84 (0.69 – 1.02) | 0.082 | 0.84 (0.59 – 1.19) | 0.321 |  |
| Manual | 0.76 (0.69 – 0.82) | <0.001 | 0.89 (0.73 – 1.07) | 0.219 |  |
| Unemployed | 0.33 (0.25 – 0.44) | <0.001 | 0.48 (0.38 – 0.61) | <0.001 |  |

RR: Risk ratio obtained from Poisson regression analyses | 95% CI: 95% Confidence Intervals

Results correspond to fully-adjusted models adjusted for all covariates in the table and accounting for regional, enumeration area and household clustering | *p* for interaction based on likelihood ratio test comparing models with and without an interaction term, with wealth and education included as continuous variables

Men n=4,458 | Women n=9,985

**Table S7** Association between sociodemographic factors and health insurance, stratified by education level

| **Sociodemographic**  **characteristics** | **No education** | | **Primary education** | | **Secondary education** | | **Higher education** | | ***p* for interaction** |
| --- | --- | --- | --- | --- | --- | --- | --- | --- | --- |
|  | **RR (95% CI)** | ***p*** | **RR (95% CI)** | ***p*** | **RR (95% CI)** | ***p*** | **RR (95% CI)** | ***p*** |  |
| **Sex** |  |  |  |  |  |  |  |  |  |
| Men | 1.00 (reference) |  | 1.00 (reference) |  | 1.00 (reference) |  | 1.00 (reference) |  | <0.001 |
| Women | 0.53 (0.28 – 0.99) | 0.048 | 0.66 (0.54 – 0.81) | <0.001 | 0.80 (0.69 – 0.93) | 0.003 | 1.02 (0.95 – 1.11) | 0.582 |  |
| **Age group** |  |  |  |  |  |  |  |  |  |
| 15-19 | ⎯ |  | 1.00 (reference) |  | 1.00 (reference) |  | 1.00 (reference) |  |  |
| 20-24 | ⎯ | ⎯ | 0.56 (0.21 – 1.48) | 0.241 | 0.52 (0.43 – 0.62) | <0.001 | 0.88 (0.60 – 1.31) | 0.534 |  |
| 25-29 | ⎯ | ⎯ | 0.76 (0.37 – 1.59) | 0.472 | 0.56 (0.46 – 0.68) | <0.001 | 1.18 (0.82 – 1.68) | 0.377 |  |
| 30-34 | ⎯ | ⎯ | 1.44 (0.64 – 3.29) | 0.381 | 0.71 (0.60 – 0.84) | <0.001 | 1.26 (0.87 – 1.83) | 0.230 |  |
| 35-39 | ⎯ | ⎯ | 1.35 (0.67 – 2.69) | 0.400 | 0.81 (0.66 – 0.99) | 0.038 | 1.19 (0.83 – 1.70) | 0.355 |  |
| 40-44 | ⎯ | ⎯ | 1.84 (0.91 – 3.71) | 0.089 | 0.86 (0.70 – 1.05) | 0.146 | 1.23 (0.92 – 1.64) | 0.157 |  |
| 45-49 | ⎯ | ⎯ | 2.51 (1.19 – 5.32) | 0.016 | 0.93 (0.73 – 1.18) | 0.533 | 1.37 (0.97 – 1.93) | 0.075 |  |
| 50-64 | ⎯ | ⎯ | 2.20 (0.99 – 4.92) | 0.054 | 0.89 (0.72 – 1.09) | 0.257 | 1.28 (0.87 – 1.87) | 0.209 |  |
| **Wealth quintile** |  |  |  |  |  |  |  |  |  |
| Lowest | 1.00 (reference) |  | 1.00 (reference) |  | 1.00 (reference) |  | 1.00 (reference) |  | 0.002 |
| Second | 2.01 (1.26 – 3.20) | 0.003 | 3.63 (1.80 – 7.34) | <0.001 | 2.05 (1.12 – 3.75) | 0.020 | 0.63 (0.08 – 4.70) | 0.647 |  |
| Middle | 5.16 (1.49 – 17.84) | 0.010 | 5.02 (2.42 – 10.41) | <0.001 | 3.49 (2.05 – 5.95) | <0.001 | 1.08 (0.37 – 3.21) | 0.885 |  |
| Fourth | 9.56 (3.19 – 27.53) | <0.001 | 8.29 (3.60 – 19.09) | <0.001 | 5.66 (3.50 – 9.16) | <0.001 | 1.53 (0.47 – 4.99) | 0.477 |  |
| Highest | 13.40 (4.66 – 38.49) | <0.001 | 9.84 (3.76 – 25.79) | <0.001 | 11.70 (7.25 – 18.89) | <0.001 | 2.00 (0.60 – 6.62) | 0.257 |  |
| **Residence type** |  |  |  |  |  |  |  |  |  |
| Urban | 1.00 (reference) |  | 1.00 (reference) |  | 1.00 (reference) |  | 1.00 (reference) |  |  |
| Rural | 0.71 (0.41 – 1.23) | 0.222 | 0.88 (0.61 – 1.27) | 0.501 | 0.99 (0.85 – 1.15) | 0.851 | 1.21 (1.07 – 1.38) | 0.003 |  |
| **Marital status** |  |  |  |  |  |  |  |  |  |
| Never married | 1.00 (reference) |  | 1.00 (reference) |  | 1.00 (reference) |  | 1.00 (reference) |  |  |
| Currently married | 3.39 (1.65 – 6.95) | 0.001 | 2.42 (1.58 – 3.70) | <0.001 | 1.92 (1.62 – 2.27) | <0.001 | 1.21 (1.07 – 1.37) | 0.003 |  |
| Living with partner | 1.27 (0.49 – 3.29) | 0.630 | 1.54 (1.07 – 2.22) | 0.020 | 1.08 (0.92 – 1.27) | 0.341 | 0.95 (0.79 – 1.15) | 0.609 |  |
| Formerly/ ever married | 1.42 (0.44 – 4.56) | 0.557 | 1.67 (0.96 – 2.91) | 0.069 | 1.11 (0.98 – 1.26) | 0.102 | 1.16 (1.05 – 1.29) | 0.004 |  |
| **Occupation** |  |  |  |  |  |  |  |  |  |
| Professional | 1.00 (reference) |  | 1.00 (reference) |  | 1.00 (reference) |  | 1.00 (reference) |  |  |
| Agricultural | 1.30 (0.78 – 2.18) | 0.321 | 0.65 (0.40 – 1.05) | 0.077 | 0.91 (0.79 – 1.06) | 0.215 | 0.94 (0.81 – 1.10) | 0.458 |  |
| Manual | 0.71 (0.52 – 0.97) | 0.031 | 1.10 (0.91 – 1.34) | 0.322 | 0.81 (0.70 – 0.93) | 0.002 | 0.69 (0.56 – 0.84) | <0.001 |  |
| Unemployed | 0.43 (0.21 – 0.91) | 0.028 | 0.28 (0.17 – 0.46) | <0.001 | 0.43 (0.35 – 0.52) | <0.001 | 0.59 (0.45 – 0.77) | <0.001 |  |

RR: Risk ratio obtained from Poisson regression analyses | 95% CI: 95% Confidence Intervals | Results correspond to fully-adjusted analyses, adjusting for all covariates in the table and regional, enumeration area and household clustering | No education n=1,213 | Primary education n=3,470 | Secondary education n=8,688 | Higher education n=1,072 | estimates not presented for age in “no education” category due to no observations in reference category | *p* for interaction based on likelihood ratio test comparing models with and without an interaction term, with wealth and education included as continuous variables

**Table S8** Association between sociodemographic factors and health insurance, stratified by wealth quintile

| **Sociodemographic**  **characteristics** | **Second wealth quintile** | | **Middle wealth quintile** | | **Fourth wealth quintile** | | **Highest wealth quintile** | | ***p*  for interaction** |
| --- | --- | --- | --- | --- | --- | --- | --- | --- | --- |
|  | **RR (95% CI)** | ***p*** | **RR (95% CI)** | ***p*** | **RR (95% CI)** | ***p*** | **RR (95% CI)** | ***p*** |  |
| **Sex** |  |  |  |  |  |  |  |  |  |
| Men | 1.00 (reference) |  | 1.00 (reference) |  | 1.00 (reference) |  | 1.00 (reference) |  | 0.012 |
| Women | 0.65 (0.42 – 1.02) | 0.061 | 0.72 (0.55 – 0.95) | 0.020 | 0.77 (0.58 – 1.04) | 0.083 | 0.89 (0.80 – 1.00) | 0.053 |  |
| **Age group** |  |  |  |  |  |  |  |  |  |
| 15-19 | 1.00 (reference) |  | 1.00 (reference) |  | 1.00 (reference) |  | 1.00 (reference) |  |  |
| 20-24 | 1.21 (0.53 – 2.78) | 0.657 | 0.95 (0.53 – 1.69) | 0.863 | 0.71 (0.54 – 0.95) | 0.019 | 0.55 (0.43 – 0.70) | <0.001 |  |
| 25-29 | 1.15 (0.48 – 2.78) | 0.755 | 1.15 (0.67 – 1.97) | 0.610 | 0.89 (0.62 – 1.28) | 0.532 | 0.57 (0.48 – 0.67) | <0.001 |  |
| 30-34 | 2.16 (0.81 – 5.77) | 0.125 | 1.15 (0.71 – 1.84) | 0.574 | 0.90 (0.67 – 1.21) | 0.486 | 0.69 (0.59 – 0.80) | <0.001 |  |
| 35-39 | 2.22 (0.72 – 6.82) | 0.164 | 1.49 (0.85 – 2.62) | 0.165 | 1.11 (0.84 – 1.47) | 0.472 | 0.70 (0.58 – 0.83) | <0.001 |  |
| 40-44 | 1.99 (0.63 – 6.29) | 0.239 | 2.12 (1.20 – 3.76) | 0.010 | 1.30 (1.03 – 1.66) | 0.029 | 0.70 (0.60 – 0.82) | <0.001 |  |
| 45-49 | 4.17 (1.29 – 13.45) | 0.017 | 2.46 (1.52 – 4.00) | <0.001 | 1.57 (1.21 – 2.04) | 0.001 | 0.74 (0.59 – 0.92) | 0.008 |  |
| 50-64 | 2.49 (0.70 – 8.82) | 0.159 | 2.09 (1.15 – 3.79) | 0.015 | 1.71 (1.25 – 2.34) | 0.001 | 0.74 (0.61 – 0.90) | 0.002 |  |
| **Education** |  |  |  |  |  |  |  |  |  |
| No education | 1.00 (reference) |  | 1.00 (reference) |  | 1.00 (reference) |  | 1.00 (reference) |  | 0.002 |
| Primary | 2.48 (1.33 – 4.61) | 0.004 | 1.47 (0.86 – 2.53) | 0.162 | 1.06 (0.78 – 1.44) | 0.699 | 0.88 (0.40 – 1.92) | 0.740 |  |
| Secondary | 4.10 (1.91 – 8.80) | <0.001 | 2.76 (1.72 – 4.43) | <0.001 | 1.67 (1.24 – 2.25) | 0.001 | 2.09 (1.18 – 3.69) | 0.011 |  |
| Higher | 9.50 (2.86 – 31.51) | <0.001 | 7.27 (3.76 – 14.05) | <0.001 | 4.19 (3.18 – 5.52) | <0.001 | 3.24 (1.81 – 5.78) | <0.001 |  |
| **Residence type** |  |  |  |  |  |  |  |  |  |
| Urban | 1.00 (reference) |  | 1.00 (reference) |  | 1.00 (reference) |  | 1.00 (reference) |  |  |
| Rural | 0.62 (0.40 – 0.97) | 0.035 | 1.06 (0.75 – 1.50) | 0.748 | 1.02 (0.86 – 1.21) | 0.805 | 1.06 (0.95 – 1.18) | 0.321 |  |
| **Marital status** |  |  |  |  |  |  |  |  |  |
| Never married | 1.00 (reference) |  | 1.00 (reference) |  | 1.00 (reference) |  | 1.00 (reference) |  |  |
| Currently married | 3.03 (2.02 – 4.55) | <0.001 | 1.97 (1.56 – 2.49) | <0.001 | 1.55 (1.27 – 1.90) | <0.001 | 1.80 (1.55 – 2.08) | <0.001 |  |
| Living with partner | 1.15 (0.71 – 1.84) | 0.571 | 0.91 (0.62 – 1.33) | 0.624 | 0.96 (0.71 – 1.31) | 0.804 | 1.19 (1.11 – 1.29) | <0.001 |  |
| Formerly/ ever married | 0.94 (0.45 – 1.99) | 0.880 | 0.99 (0.73 – 1.35) | 0.954 | 1.04 (0.77 – 1.42) | 0.783 | 1.29 (1.09 – 1.52) | 0.003 |  |
| **Occupation** |  |  |  |  |  |  |  |  |  |
| Professional | 1.00 (reference) |  | 1.00 (reference) |  | 1.00 (reference) |  | 1.00 (reference) |  |  |
| Agricultural | 1.00 (0.47 – 2.10) | 0.989 | 1.19 (0.81 – 1.77) | 0.372 | 0.62 (0.42 – 0.91) | 0.016 | 0.90 (0.78 – 1.03) | 0.126 |  |
| Manual | 0.94 (0.50 – 1.76) | 0.842 | 0.86 (0.68 – 1.10) | 0.227 | 0.77 (0.56 – 1.06) | 0.108 | 0.87 (0.77 – 0.98) | 0.026 |  |
| Unemployed | 0.19 (0.11 – 0.34) | <0.001 | 0.32 (0.23 – 0.44) | <0.001 | 0.30 (0.23 – 0..38) | <0.001 | 0.60 (0.49 – 0.73) | <0.001 |  |

RR: Risk ratio derived from Poisson regression analyses | 95% CI: 95% Confidence Intervals | Results correspond to fully-adjusted analyses, adjusting for all covariates in the table and regional, enumeration area and household clustering | N.B. Lowest Wealth Quintile not shown due to non-convergence of model | Second quintile n=2,678 | Middle quintile n=3,048 | Fourth quintile n=3,381 | Highest quintile n=3,035 |  *p* for interaction based on likelihood ratio test comparing models with and without an interaction term, with wealth and education included as continuous variables
